# The Association of the Colleges of Dentistry

**Published:** 1867-04

**Authors:** 


### THE ASSOCIATION OF THE COLLEGES OF DENTISTRY.

The Association of the Colleges of Dentistry met in the lecture room of the Philadelphia Dental College, in Philadelphia, on Wednesday, March 20th, 1867, at 10 o'clock, A. M.

Professor E. Parmley, President in the Chair.

There were present from the Baltimore Dental College, Professors P. H. Austin, F. J. S. Gorgas; Ohio Dental College, Prof. J. Taft; Penn. Dental College, Prof. T. L. Buckingham, George T. Barker, E. Wildman, W. S. Forbes, J. Truman;

Philadelphia Dental College, J. H. McQuillen, J. F. Flagg, Thomas Wardle, C. A. Kingsbury, J. E. Garretson, Lecturer of Clinical Surgery; New York Dental College, Prof. E. Parmley, F. D. Weisse, N. W. Kingsley, R. King Brown.

The minutes of the last meeting were read and approved.

The name of Prof. Judd, of the Missouri College of Dentistry, was presented for membership in this Association.

A committee of three was appointed to receive and report upon the application.

Committee consisted of Profs. George T. Barker, N. W. Kingsley and J. H. McQuillen.

After due deliberation, the committee made the following report:

We have examined the credentials of Prof. H. Judd as a delegate to this Association, and would respectfully report that owing to the peculiar position in which the institution now stands that he represents, we do not feel at liberty to recommend him as a member of this body, but would suggest that for the present he be invited to be present at the sessions of this meeting, and take part in the deliberations.

Signed, George T. Barker, N. W. Kingsley, J. H. McQuillen, } *Com.*

On motion of Prof. Weisse,

*Resolved*, That the resolutions presented, considered and approved at the last meeting of this Association be now taken up and adopted; after which, the following resolutions constituting regulations for all the Colleges represented in this body were discussed, pending which the Association adjourned to meet in the lecture room of the Penn. Dental College, at 3½ o'clock, P. M.

AFTERNOON SESSION.

The Association met at 4½ o'clock, P. M., at the place designated in the adjournment.

Minutes of the morning session were read and approved.

The following regulations and by-laws were now adopted:

I. That the rule of our Dental Colleges allowing one session in a medical college to be considered equivalent to one course in a Dental College be abolished.

II. That two full years of pupilage with a reputable Dental practitioner, inclusive of two complete courses of lectures in a Dental College, be required to entitle the candidate to an examination for graduation for the degree of D. D. S.

III. That a graduate of a respectable medical college, who has been under the pupilage of a reputable Dentist for one year, and shall have attended one full course of lectures in a Dental College, shall be entitled to examination for the degree of D. D. S.

IV. That eight years of Dental practice, including regular pupilage, will be regarded as equivalent to one course of lectures.

V. That the regular term of instruction in the Dental Colleges, be five months, the sessions in each to commence on the third Monday of October, annually.

VI. That students entering the Colleges later than the 10th of November, will not be credited for a full course, nor be eligible to graduation at the same term.

VII. That a candidate for graduation will be required to furnish a written certificate of having pursued the required pupilage, or period of practice.

VIII. Regarding the education of the profession as the primary and only object in the establishment of Dental Colleges, therefore,

*Resolved*, That whilst this Association does not forbid, it cannot approve the conferring of degrees upon persons who have not complied with the regulations agreed upon by this body, with the exception of gentlemen who have distinguished themselves as contributors to Dental science.

The regulation marked number eight was *very* warmly and earnestly discussed by almost all the members; pending which, a motion was made by Prof. George T. Barker to lay it on the table. The vote upon this motion being taken by Colleges was as follows:

Yea, Penn. Dental College.

Nay, Baltimore Dental College,
" Ohio " "
" Philadelphia " "
" New York " "

After some further discussion, and amendment of the resolution by Prof. Austin, the vote upon it was taken, and was as follows:

Yea, Baltimore Dental College,
" Ohio " "
" Philadelphia " "
" New York " "
Nay, Penn. " "

Immediately after this vote, the Faculty of the Penn. Dental College announced through their Dean that the passage of this resolution rendered it necessary for them to withdraw from this Association, alleging for this movement their conviction that it is a rebuke upon their past practice of conferring degrees upon practitioners of Dentistry, and also a restriction upon their intended future course in this respect.

Adjourned until 9 o'clock to-morrow morning.

### SECOND DAY, MORNING SESSION.

Thursday morning, 9 o'clock.

Association met according to appointment, in the lecture room of the Philadelphia Dental College.

The minutes of the last meeting were read and approved.

Dr. James E. Garretson presented the following:

*Resolved*, That we recognize that the truest dignity of the Dental, as any other specialty, is found alone in the education of its practitioners, and that this education should be one common to all medical men, and that it be the object of the Association of Colleges to so educate their students—advancing to this object as rapidly as circumstances seem to warrant, thus merging the specialty into the common mother practice.

This resolution called forth some earnest discussion, after which, the following was submitted by Prof. R. King Brown:

Honoring the sentiments which animate Dr. Garretson in the presentation of his resolution, and favoring the fullest and most ample instruction on the part of Dental Colleges, but considering that a matter which should be left to the different Faculties, I respectfully move that the resolution be laid upon the table.

On motion of Prof. Weisse:

*Resolved*, That we re-consider the action of yesterday, in regard to the reception of Prof. H. Judd, of the Missouri Dental College as a member of this Association.

The vote was unanimous for the reconsideration.

On motion of Prof. Weisse:

*Resolved*, That on the establishment in the Missouri Dental College of such additional chairs as are regarded by this Association necessary to qualify Dental practitioners, viz: those of Operative and Mechanical Dentistry, the said Faculty shall become *ipso facto* members of this Association.

On motion of Dr. McQuillen:

*Resolved*, That the next annual sessions of the Colleges begin on the 15th day of October, 1867.

On motion of Dr. Kingsley:

*Resolved*, That when this Association adjourn, it be to meet in the city of New York, on the 19th day of March, 1868.

It was moved that the expenses of the Association be paid by an assessment on the Colleges.

On motion of Dr. Weisse:

*Resolved*, That the Secretary be requested to have the Constitution, By-Laws and Regulations of this Association stereotyped, for printing sheets for distribution to the different Faculties.

J. Taft was appointed Treasurer of the Association.

A vote of thanks was tendered to the Faculties of the Dental Colleges of Philadelphia for the courtesy and kindness received at their hands by this Association during its sessions here.

The various Faculties, in the person of their various official officers, signed the Constitution and By-Laws, after which the Association adjourned to meet in the city of New York on the 19th of March, 1868.

J. TAFT, *Secretary.*

---

The second annual meeting of the Central Ohio Dental Association will be held at Galion, on the 14th, 15th and 16th of May next.

A. W. MAXWELL, *Secretary.*

